# Sex-specific differences in recurrence and progression following cytostatic intravesical chemotherapy for non-muscle invasive urothelial bladder cancer (NMIBC)

**DOI:** 10.1007/s00432-025-06108-x

**Published:** 2025-02-01

**Authors:** Laila Schneidewind, Bernhard Kiss, Thomas Neumann, Jennifer Kranz, Friedemann Zengerling, Sebastian Graf, Annabel Graser, Annemarie Uhlig

**Affiliations:** 1 Department of Hematology/Oncology, Ferdinand-Sauerbruchstr, University Medical Center Greifswald, 17475 Greifswald, Germany; 2 Department of Urology, University Hospital of Bern, Bern, Switzerland; 3 Department of Urology and Pediatric Urology, University Medical Center RWTH Aachen, Aachen, Germany; 4 Department of Urology and Kidney Transplantation, Martin Luther University, Halle (Saale), Germany; 5 Department of Urology and Pediatric Urology, University Hospital Ulm, Ulm, Germany; 6 Department of Urology and Andrology, Kepler University Hospital Linz, Linz, Austria; 7 Department of Urology, Ludwig Maximilian University, Munich, Germany; 8 Department of Urology, University Medical Center Göttingen, Göttingen, Germany

**Keywords:** Bladder cancer, Urothelial cancer, MIBC, Intravesical chemotherapy, Gender, Sex

## Abstract

**Purpose:**

To systematically analyze gender-specific differences in recurrence-free survival (RFS), progression-free survival (PFS), cancer-specific survival (CSS), and overall survival (OS) as well as adverse events and quality of Life (QoL) as secondary aims in NMIBC patients undergoing cytostatic intravesical chemotherapy.

**Methods:**

A systematic review and meta-analysis were conducted on studies published between 1976 and 2024, following PRISMA guidelines. MEDLINE, Embase and Cochrane Library were used as literature sources. No restrictions were made concerning language, study region or publication type. Data from 12 studies encompassing 1,527 patients were analyzed. Outcomes were assessed using random-effects models, with gender as a primary variable of interest. A risk of bias assessment was done using the ROBINS-I tool or RoB2 as appropriate.

**Results:**

The pooled analysis demonstrated no statistically significant gender-specific differences in RFS (HR = 1.0625, 95% CI 0.8094–1.0526) or PFS (HR = 1.0861, 95% CI 0.7038–1.6760). Data on CSS and OS were insufficient for meaningful conclusions. Two included studies analyzed in univariate or multivariate regression gender as risk factor for recurrence or progression, but gender was not a significant risk factor. Adverse events and QoL outcomes were notably underreported, with no gender-specific data available.

**Conclusions:**

While this study found no significant gender-based differences in NMIBC outcomes following intravesical chemotherapy, the findings are limited by the small number of studies, underrepresentation of women, and inconsistent reporting of critical outcomes. Future research should prioritize gender-focused analyses and explore the molecular and genetic basis of potential differences to inform precision medicine and equitable care.

**Supplementary Information:**

The online version contains supplementary material available at 10.1007/s00432-025-06108-x.

## Introduction

Bladder cancer (BC) is the seventh most commonly diagnosed cancer in men worldwide, and ranks tenth when both genders are considered. The global age-standardized mortality rate per 100,000 person-years is 3.3 for men and 0.86 for women (International Agency for Research on Cancer. Estimated number of new cases in 2020; EAU 2024). Approximately 75% of BC cases are non-muscle-invasive (NMIBC), confined to the mucosa (stage Ta, CIS) or submucosa (stage T1). This percentage is even higher in younger patients (< 40 years of age) (EAU 2024; Comperat et al. 2015). Patients with NMIBC have a high disease prevalence due to long-term survival in many cases (International Agency for Research on Cancer 2020; EAU 2024; Burger et al. 2013). Due to a considerable risk of recurrence and progression NMIBC patients require a life-long follow-up, rendering NMIBC one of the most cost-intensive malignancies worldwide (EAU 2024).

Based on the risks of recurrence and progression, the European Association of Urology (EAU) stratifies NMIBC into four risk groups Intermediate-risk NMIBC includes patients without CIS, who do not fall into the low-, high-, or very high-risk groups. These patients have a 10-year probability of progression of about 3.7% (95% CI 2.3% − 5.9) and a considerable risk of recurrence depending on the presence of risk factors: The 1-year probability of recurrence ranges from 19.5 to 61% with 0 or ≥ 3 risk factors. According to the EAU guideline repeat chemotherapy instillations (with or without previous single postoperative instillation) improve recurrence-free survival (RFS) in intermediate-risk patients. However, the optimal duration and frequency of repeat chemotherapy instillations remain controversial, but it should not exceed one year. In high-risk NMIBC patients the risk for recurrence and progression, as it is self-explanatory, are even higher. Therefore, EAU guideline recommends Bacillus Calmette–Guérin (BCG) instillation therapy with a 3-year maintenance scheme following induction. However, due to the ongoing global BCG shortage, intravesical chemotherapy is often used as an alternative.

There are hints from trials, literature as well as from our working group, that there are significant gender-specific differences in response to urothelial bladder cancer therapy. However, these data are still inconclusive and require a very detailed reflection (EAU 2024; Bilski et al. 2022; Fadel et al. 2022; Uhlig et al. 2018). Understanding of these differences has the potential to inform individualized precision medicine and improvement patient outcomes.

Therefore, this study aims to address the question: “Are there gender-specific differences in recurrence and progression following cytostatic intravesical chemotherapy for intermediate and high-risk NMIBC?” The primary aim of this study is to evaluate gender specific differences in RFS, progression free (PFS), cancer specific (CSS), and overall survival (OS). The secondary aim is to assess gender-specific differences in adverse events and quality of life (QoL).

## Material and methods

### Search strategy

In May 2024, we performed a systematic literature search using MEDLINE via PubMed, Embase, and the Cochrane Library. The search algorithm broadly included the search term clusters gender, bladder cancer, NMIBC, outcomes and intravesical chemotherapy. The supplementary material (Supplementary 1) details the complete search algorithms. Reference lists of included articles, as well as review articles, were searched to identify additional records. No restrictions were made concerning language, study region, or publication type. Studies published after January 1976 were included, as this marks the introduction of the first intravesical BCG-instillations for NMIBC. This study was prospectively registered at PROSPERO (https://www.crd.york.ac.uk/prospero/; ID CRD 42024507059).

### Study inclusion and exclusion criteria

The predefined primary outcomes were gender-specific differences in RFS, PFS, CSS and OS following single intravesical instillation therapy with cytostatic intravesical chemotherapy in intermediate- and high-risk NMIBC. No restrictions were made regarding the choice of cytostatic agents. We included randomized controlled trials (RCTs), prospective clinical trial (non-RCTs), and retrospective cohort studies. Combination therapies involving radiotherapy, chemotherapy or other targeted therapies were excluded. Furthermore, patients with a history of prior intravesical therapies within six months before initiation of the study therapy were excluded to minimize potential confounding effects. Additionally, we focused on urothelial histological subtype. If multiple publications evaluated the same patient cohort, the larger and more comprehensive publication was included. Exclusions were made according to the registered PROSPERO protocol.

### Data extraction

An a priori defined standardized data extraction process was used for every included record. Extracted variables included author(s), year of publication, study country, population size, percent of female patients, cancer stage and grade, histopathological cancer subtype, length of follow-up, details on chemotherapy as well as dosing in the included studies, variables adjusted for in multivariable Cox regression models and HR or OR measures with the associated 95% CI for RFS, PFS, CSS and OS. Study extraction was independently performed by two review authors. Inconsistencies were resolved by a third review author. The online platform covidence (https://www.covidence.org/; Veritas Health Innovation Ltd, Melbourne, Australia) was used for the screening and data extraction process.

### Study quality assessment

Two reviewers independently assessed the risk of bias with the ROBINS-I-tool or the Cochrane Risk of Bias tool RoB2 as appropriate (Cohrane Germany 2021). The ROBINS-I tool includes seven domains of bias: risk of bias due to confounding, bias in the selection of participants into the study, bias in classification of interventions, bias due to deviations from intended interventions, bias due to missing data, bias in the measurement of outcomes and bias in the selection of the reported results for one outcome measurement. The domains are combined to an overall risk of bias. The RoB2 tool summarizes five risk of bias domains: bias arising from randomization process, bias due to deviations from the intended intervention, bias due to missing outcome data, bias in measurement of the outcome and bias in selection of the reported results. These domains are also combined to an overall risk of bias. Any disagreements were resolved by the involvement of a third review author.

### Statistical analysis

Comparison of gender-specific differences in survival parameters was performed using the inverse variance method weighting for pooling of continuous outcome data to account for clinical heterogeneity (Shu et al. 2021). In all provided analyses, male patients were considered the referent. Studies providing estimates with a female referent were back-calculated by inversing the hazard ratios (HR) and the associated confidence intervals (CIs). Between studies, heterogeneity was assessed by the I^2^ statistic with the associated 95% CI, the chi-square p-values of heterogeneity and visual inspection of forest plots. Heterogeneity was interpreted as limited—I^2^ = 0–40%, moderate—I^2^ = 41 to 60%, substantial—I^2^ = 61 to 80% and considerable I^2^ = 81 to 100%. In addition, change of pooled HR over the years of publication was assessed. Publication bias was assessed by visual inspection of the funnel plot. All statistical analyses were performed with R version 4.2.1 (https:// www.r-project.org/) and RStudio (RStudio, Boston, Massachusetts) and the R package meta (Schwarzer 2007). The alpha level indicating statistical significance was predefined as 0.05 for all analyses except the assessment of heterogeneity, which was considered at alpha = 0.1. All provided p-values are 2-sided.

## Results

### Study characteristics

**Fig. 1:**
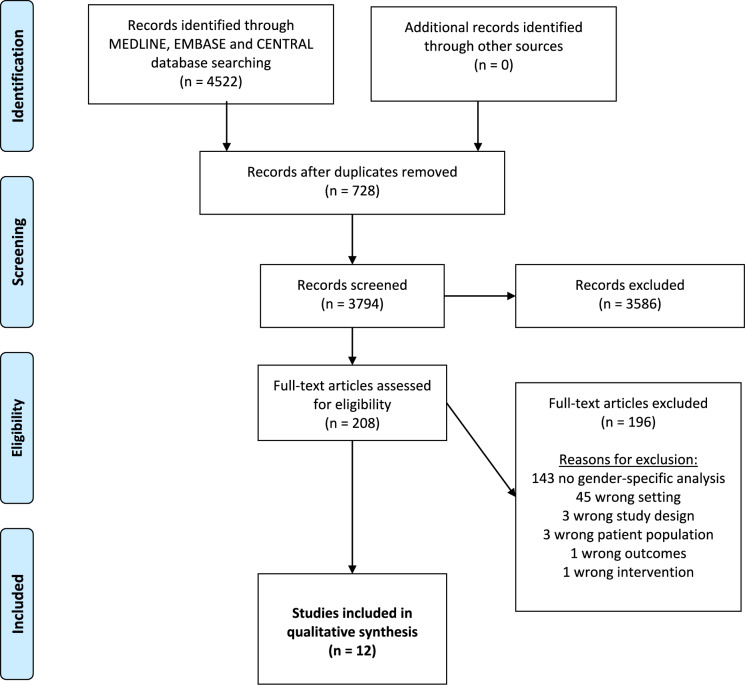
PRISMA flowchart

The systematic literature search identified 4,522 studies of which 12 fulfilled the inclusion criteria and reporting about 1,527 patients. The PRISMA (Preferred Reporting Items for Systematic Reviews and Meta-Analyses) flowchart is shown in Fig. 1. The most frequent reason for exclusion in the full text screening was “no gender specific analysis” (n = 143). The characteristics of the included studies are summarized in Table 1.

**Table 1:** Summary of study characteristics (*n* = 12)

References	Study design	Number of participants	Percentage of females	Evaluation of gender-specific differences as study aim	Chemotherapy used	Induction/maintenance	Outcomes	Median length of follow-up (months)
Bono et al. 1994	Cohort retrospective, monocentric	145	15.0	no	Doxorubicin	induction and maintenance	RFS; PFS; OS	73.1
Chen et al. 2012	Cohort retrospective, monocentric	290	27.8	no	Doxorubicin alone versus BCG alone versus sequential of Mitomycin, doxorubicin and cisplatin	induction and maintenance	RFS; Adverse events; Other: progression or metachronous upper tract carcinoma	50.0
Hurle et al. 1998	Cohort retrospective, monocentric	242	23.1	no	Mitomycin	induction and maintenance	RFS; PFS; OS; CSS	57.0
Kato et al. 2015	Cohort retrospective, monocentric	90	18.9	no	Epirubicin plus Cytarabine-C	induction and maintenance	RFS; PFS; Adverse events	33.7 (mean)
Koga et al. 2004	Randomized controlled trial, prospective multicentric	150	26.7	no	Epirubicin	induction and maintenance	RFS; Adverse events	30.6
Kondo et al. 1999	Cohort retrospective, monocentric	45	20.0	no	Epirubicin	induction and maintenance	RFS; Adverse events; Other: Prognostic factors	36.7 (mean)
Maeda et al. 2011	Cohort retrospective, monocentric	124	25.0	no	Mitomycin	induction and maintenance	RFS; Adverse Events	30.0
Matsumura et al. 1986	Cohort prospective monocentric	35	57.0	no	Epirubicin	induction and maintenance	Other: Response rate	NN
Mitsumori et al. 2004	Cohort prospective, multicentric	69	26.1	no	Epirubicin	induction and maintenance	RFS; Adverse events	13.3
Wang et al. 2019	Cohort retrospective, monocentric	124	26.6	no	Gemcitabine versus Epirubicin or Pirabucin	induction and maintenance	RFS; PFS; Adverse events	35.0 (mean)
Wang et al. 2022	Cohort prospective, monocentric	160	23.1	no	Pirarubicin, Pharmorubicin and Gemcitabine	induction and maintenance	RFS; PFS	81.4
Watanabe et al. 1994	Cohort prospective, monocentric	53	60.0	no	Epirubicin	induction and maintenance	Other: recurrence rates	9.25 (mean)

*RFS* Recurrence free survival, *PFS* Progression free survival, *OS* Overall survival, *CSS* Cancer specific survival

All studies were published in English language between the years 1986 and 2022 and included mainly patients with urothelial subtype NMIBC (two studies did not report the histological subtype and one study included three patients with other histological subtype of NMIBC) and only receiving cytostatic intravesical chemotherapy. Furthermore, all included studies were full published articles. Only two studies were designed in a multicenter setting and only one was a randomized controlled trial whereas the remainder were cohort studies. Four of the cohort studies had a prospective setting. The study regions mainly included Asia, solely two investigations were conducted in Europe.

Additionally, none of the included studies had gender-specific differences as study aim, but three studies (25%) used multivariate models with gender as covariate. The study size ranged from 35 to 290 patients and included 15.0 to 60.0% female participants. Median/mean patient age ranged from 62 to 68 years (available for 8 studies, 66.7%). The intravesical chemotherapy used was mainly epirubicin (in seven studies, 58.3%), but in various combinations and very different applicant schemes. All patients received induction as well as maintenance therapy.

The outcomes evaluated were RFS (10 studies), PFS (5 studies), OS (2 studies), CSS (1 study), adverse events (7 studies, but no gender-specific differences reported) and others (3 studies). The length of follow-up, if documented ranged from 9 to 73 months (available for 11 studies, 91.7%). Pooling was possible for the outcomes RFS and PFS.

### Pooled analysis for RFS

**Fig. 2:**
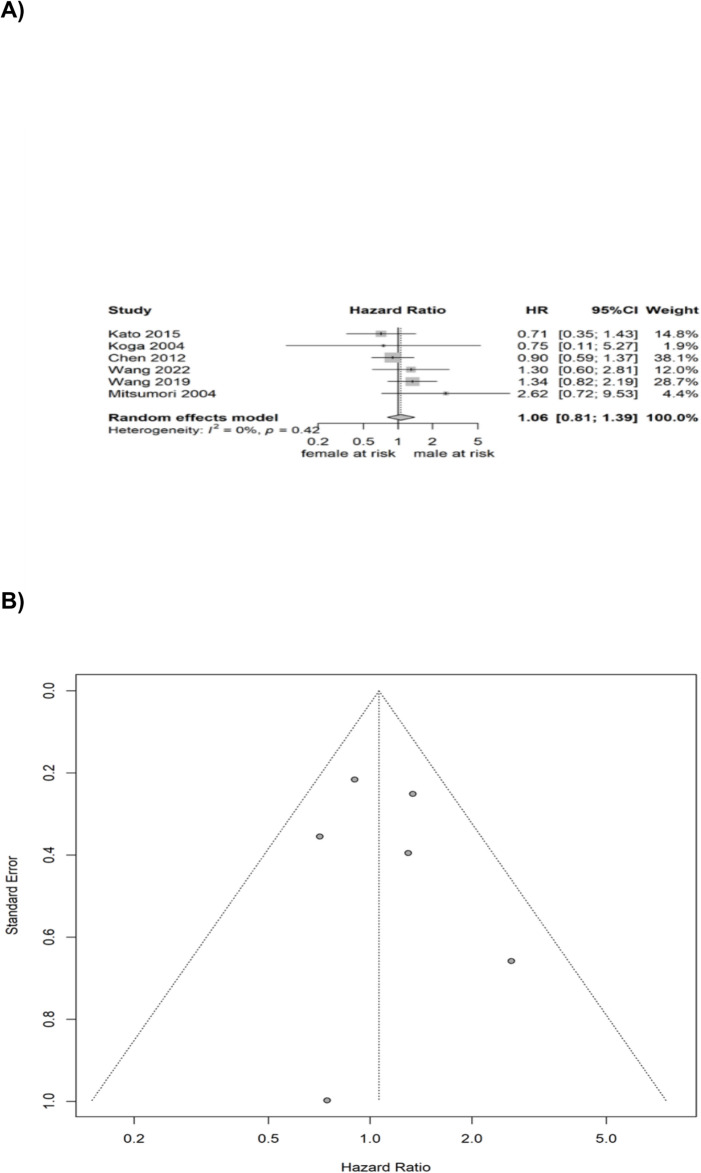

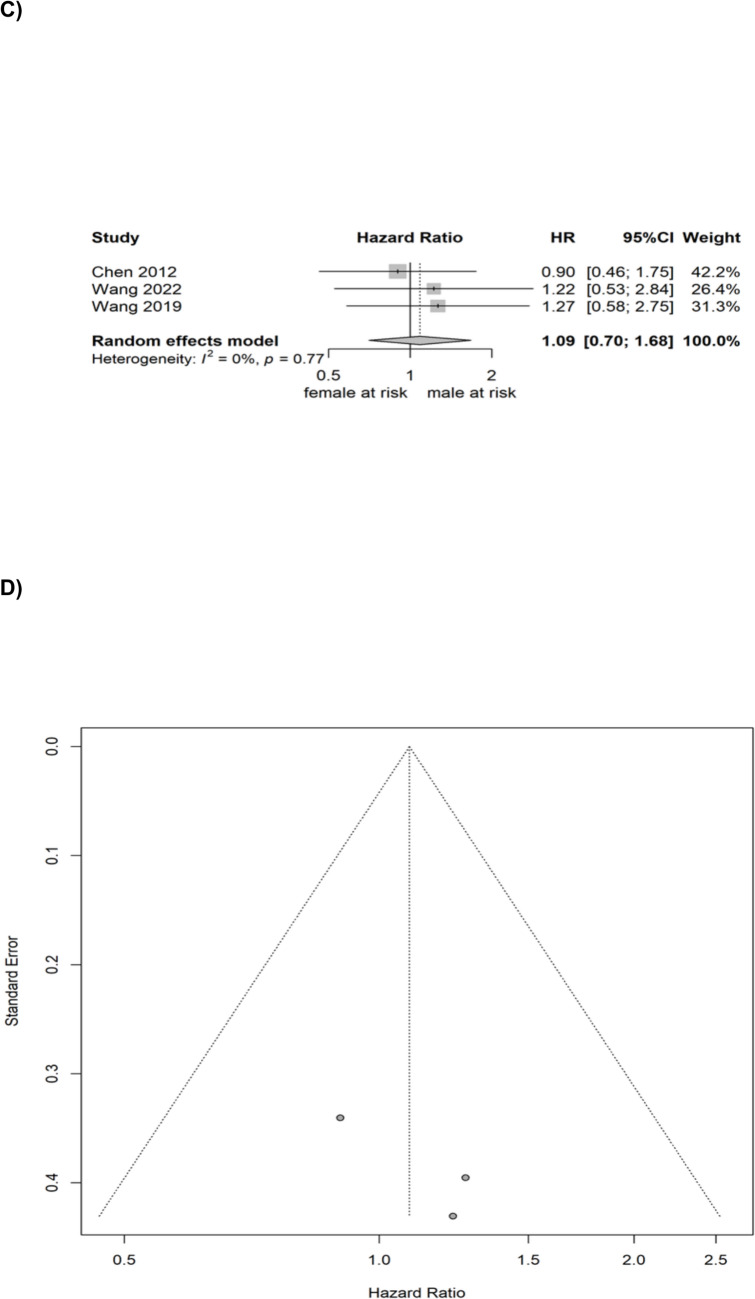
Pooled analysis for RFS and PFS—**A** Forrest plot of the random-effect meta-analysis for RFS; **B** Funnel plot for the pooled studies for RFS; **C** Forrest plot of the random-effect meta-analysis for PFS; **D** Funnel plot for the pooled studies for PFS

Random effect meta-analysis for RFS included data from 6 studies and yielded no statistically significant gender-specific difference (HR comparing males to females = 1.0625, 95% CI: 0.8094–1.0526, p = 0.6622). Heterogeneity was limited (I^2^ = 0.0%; p = 0.4195). No publication bias was detected upon visual inspection. This analysis is illustrated in Fig. 2.

### Pooled analysis for PFS

Random effect meta-analysis for PFS included data from 3 studies and yielded no statistically significant gender-specific difference (HR comparing males to females = 1.0861, 95% CI: 0.7038–1.6760, p = 0.7091). Heterogeneity was limited (I^2^ = 0.0%; p = 0.7669). No publication bias was detected upon visual inspection. This analysis is also illustrated in Fig. 2.

### Non-pooled outcome parameters

Two studies reported data about OS, but no gender-specific differences were reported (Bono et al. 1994; Hurle et al. 1998). Additionally, Hurle et al. investigated CSS, but reported no sex specific details, too. However, they reported in multivariate analysis risk factors for treatment failure and disease progression, sex was not significant in both, p = 0.8844 and p = 0.4695, respectively (Hurle et al. 1998). Chen et al. analyzed data about progression or metachronous upper tract carcinoma, but they also give no details about gender differences (Chen et al. 2012). Additionally, Kondo et al. described in univariate analysis prognostic factors for 2-years and 5-years recurrence free rates. Gender is not a significant risk factor in this analysis, p = 0.725 (Kondo et al. 1999). Nearly the same was reported by Matsamuro et al. and by Watanabe et al. with also no gender-specific differences in response rates or recurrence rates, respectively (Matsumura et al. 1986; Watanabe et al. 1994).

### Secondary study aims

None of the included studies reported data about gender-specific differences in adverse events and QoL.

### Quality assessment

**Fig. 3:**
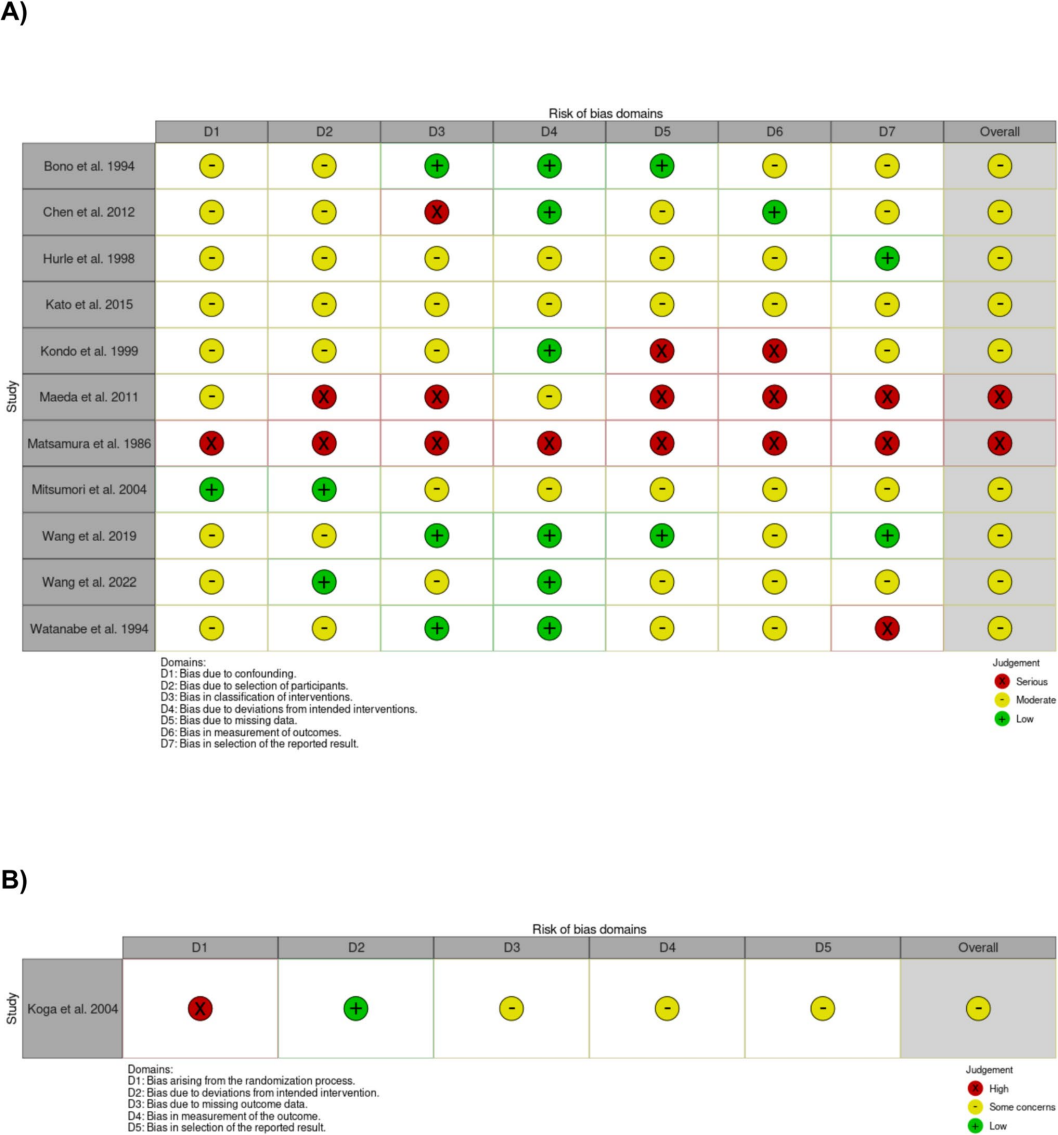
Detailed risk of bias/quality assessment—**A** Cohort studies with ROBIN-I tool; **B** Randomized controlled study with RoB2 tool

The risk of bias assessment yielded an overall moderate to high risk of bias. Reason for limited quality was mainly due to the retrospective study designs. Additionally, there were high risk of bias due to missing data and in measurement of outcomes. Figure 3 shows the detailed risk of bias assessment of the included studies.

## Discussion

Gender-specific differences in bladder cancer have been well-documented, with women often diagnosed at more advanced stage and exhibiting worse overall prognosis (Radciewicz et al. 2020). However, for NMIBC, evidence regarding female gender as a negative prognostic factor remains inconclusive (Bilski et al. 2022). This study addresses this question within the context of intravesical chemotherapy for NMIBC through a comprehensive systematic review and meta-analysis.

The pooled analysis revealed no significant gender-specific differences for RFS (HR = 1.0625, p = 0.6622) or PFS (HR = 1.0861, p = 0.7091). Furthermore, no statistically significant gender-specific differences were evident in individual studies. However, instead of accepting these results as evidence of gender neutrality in intravesical chemotherapy outcomes, they must be critically evaluated: First, the available literature demonstrated serious study limitations. Second, if there are suspected gender-specific differences, underlying reasons and mechanisms of the differences, but also the reasons for them not being evident in this meta-analysis, must be discussed.

The number of studies adequate for inclusion in our meta-analysis was relatively small. Especially the pooled results rely on six studies for RFS and even three studies for PFS. None of the studies was specifically designed to assess gender-specific differences. Critical outcomes, including CSS and OS, were inconsistently reported, further limiting the ability to draw robust conclusions. Furthermore, none of the studies explicitly aimed to investigate sex-specific differences, and only three (25%) included gender as a covariate in multivariate models, leaving a significant research gap in addressing this question explicitly. Moreover, the sample size and representation in the studies can be criticized: The included studies had relatively small sample sizes and low representation of women (15–60% female participants). This limits the statistical power to detect potential differences. Even though the follow-up was relatively long (mean/median > 30 months in 70% of the studies) the results are only estimations about long-term disease recurrence or progression.

In addition, chemotherapy regimens were rather variable: The studies utilized diverse chemotherapy agents and dosing schedules, which could have introduced clinical heterogeneity that was not fully accounted for despite the use of random-effects models.

Another flaw is that none of the included studies reported gender-specific data on adverse events or quality of life aspects. These outcomes are essential for assessing the full scope of treatment impacts, particularly given known differences in side effects between genders (Unger et al. 2022).

Furthermore, the reporting of risk stratification as well as concomitant carcinoma in situ was very poor. There were also different risk definitions used. No gender specific analysis on this issue was possible, but the risk stratification must be considered for identification of sex specific patterns and should be consequently addressed in further investigations.

Lastly, the risk of bias of the studies was rated as moderate to high risk primarily due to missing data and potential measurement errors. These factors reduce the reliability of the pooled analyses. In summary, even though no differences were evident, they might be fogged by the shortcomings of the available literature. In this context it must also be discussed that most studies were conducted in Asia. Leading to the questions, if ethnicity and the genetics behind it, play a crucial role in response to NMIBC treatments. Consequently, this should be addressed in further robust clinical evaluations.

Despite these limitations, the findings align with previous work from our group, which also found no gender-specific differences in recurrence rates for NMIBC patients receiving intravesical chemotherapy with or without bacillus Calmette-Guérin (BCG) (Uhlig et al. 2018; Schneidewind et al. 2024). This consistency may suggest that intravesical chemotherapy efficacy is not inherently influenced by gender, at least for intermediate- and high-risk NMIBC patients. Still, the differences of chemotherapy or immunotherapy or their combinations must be considered and might explain controversial results in the literature (Uhlig et al. 2018).

These results are interesting in the light of women being known to suffer from more advanced disease upon diagnosis (Mallin et al. 2011; Dobruch et al. 2016; Cárdenas-Turanzas et al. 2006; Danforth et al. 2020; Hasan et al. 2022). With respect to disease recurrence and progression among NMIBC patients the literature is inconclusive (Kluth et al. 2013; Palou et al. 2012; Lobo et al. 2024). Yet, research on urothelial carcinoma biology has proposed various mechanisms entailing potential differences. As an example, differences in hormone receptor expression may affect tumor biology and response to treatment. Furthermore, it is known that women and men have different immune responses to the same, e.g. infectious stimuli. Interestingly, regulatory B cells have an anti-inflammatory effect. Additionally, older studies have shown that oestrogens can upregulate regulatory B cells and thus indirectly have an anti-inflammatory effect. Some authors conclude from this finding that auto-immune-mediated diseases, such as multiple sclerosis, improve clinically or do not progress during pregnancy (Bodhankar et al. 2011, 2012; Chenard et al. 2021; Guzman-Genuino and Diener 2017; Jones et al. 2016, 2019; Sarvaria et al. 2017). Consequently, it can now be assumed that the anti-inflammatory regulatory B cells are more highly expressed in women than in men due to the presence of oestrogen and that the anti-inflammatory effect makes a difference in controlling NMIBC.

However, sex-specific differences in OS could also be searched elsewhere, e. g. in outcomes of early radical cystectomy after BCG failure. Therefore, started a new systematic review and meta-analysis called: “Gender-specific differences in outcome and survival of early cystectomy in patients who failed BCG instillation therapy for bladder cancer” (https://www.crd.york.ac.uk/prospero/; ID CRD 42024611111).

In summary, gender differences have been documented in other contexts. This raises the possibility that such differences may exist for intravesical cytostatic chemotherapy but remain underexplored due to the limited focus on gender in the included studies. Therefore, it is unclear whether current treatment protocols for intermediate- and high-risk NMIBC do require modification based on gender. This calls for prospective assessment of gender as a potential confounder in future studies with more diverse patient populations from all over the world. A comprehensive reporting of adverse events, QoL and patient-reported outcomes should be integral to study designs to provide a holistic understanding of treatment impact and guide patient-centered care.

Future research should also explore molecular and genetic mediators of gender-specific chemotherapy response and could illuminate subgroup-specific effects, potentially guiding personalized therapies.

## Conclusion

This study provides thorough research of the available literature about the role of gender in NMIBC outcomes following cytostatic intravesical chemotherapy. While no significant gender-specific differences in RFS and PFS were found among NMIBC patients receiving intravesical chemotherapy, these findings are limited by the quality and scope of the available evidence. Comprehensive, gender-focused research is essential to clarify whether these results reflect genuine equality in outcomes or are masked by study limitations. This remains crucial to ensuring equitable and effective care for all NMIBC patients.

## Supplementary Information

Below is the link to the electronic supplementary material.

Supplementary file1 (DOCX 21 KB)

## Data Availability

No datasets were generated or analysed during the current study.
