# Supplementary material for: Sex-specific differences in recurrence and progression following cytostatic intravesical chemotherapy for non-muscle invasive urothelial bladder cancer (NMIBC)
Source: J Cancer Res Clin Oncol. 2025 Feb 1;151(2):59. doi: 10.1007/s00432-025-06108-x (PMC11787168; doi:10.1007/s00432-025-06108-x)
Supplement: Supplementary file 1 — Supplementary file1 (DOCX 21 KB) [file 432_2025_6108_MOESM1_ESM.docx]

**Supplementary 1 – Search Strategies (Last search 11.04.2024)**

**MEDLINE via PubMed**

(“Urinary Bladder Neoplasms”[Mesh] OR “Carcinoma, Transitional Cell”[Mesh] OR (Bladder Cancer*) OR (Bladder Tumor*) OR (Bladder Tumour*) OR (Bladder Neoplas*) OR (Urothelial Cancer*) OR (Urothelial Carcinoma*) OR (Urothelial Tumor*) OR (Urothelial Tumour*) OR (Urothelial Neoplas*) OR (Transitional Cell Carcinoma*))

AND

(“Administration, Intravesical”[Mesh] OR (Bladder Instillation Therap*) OR (Bladder Immune Instillation Therap*) OR (Intravesical Instillation) OR (Intravesical Chemotherap*))

AND

(“Female”[Mesh] OR “Male”[Mesh] OR “Sex”[Mesh] OR “Women”[Mesh] OR “Men”[Mesh] OR (Gender) OR (Man) OR (Men) OR (Woman) OR (Women) OR (Male*) OR (Female*) OR (Sex))

AND

(“Neoplasm Recurrence, Local”[Mesh] OR “Recurrence”[Mesh] OR “Treatment Failure “[Mesh] OR “Disease Progression”[Mesh] OR “Treatment Outcome”[Mesh] OR “Survival”[Mesh] OR “Prognosis”[Mesh] OR (Recurrence) OR (Recurrent Disease) OR (Treatment Failure) OR (Disease Progression) OR (Treatment Outcome*) OR (Survival) OR (Prognosis) OR (Disease Progression) OR (Disease Exacerbation))

**Embase**

| 1 | exp bladder tumor/ |
| --- | --- |
| 2 | exp transitional cell carcinoma/ |
| 3 | (bladder cancer* or bladder tumor* or bladder tumour* or bladder neoplas* or urothelial cancer* or Urothelial Carcinoma* or urothelial tumor* or urothelial tumour* or urothelial neoplas* or transitional cell carcinoma*).tw,kw. |
| 4 | 1 or 2 or 3 |
| 5 | exp intravesical drug administration/ |
| 6 | (Bladder instillation therap* or Bladder immune instillation therap* or Intravesical instillation or intravesical chemotherap*).tw,kw. |
| 7 | 5 or 6 |
| 8 | exp female/ |
| 9 | exp male/ |
| 10 | exp sex/ |
| 11 | exp sex/ |
| 12 | (gender or man or men or woman or women or male* or female* or sex).tw,kw. |
| 13 | 8 or 9 or 10 or 11 or 12 |
| 14 | exp tumor recurrence/ |
| 15 | exp recurrent disease/ |
| 16 | exp treatment failure/ |
| 17 | exp disease exacerbation/ |
| 18 | exp treatment outcome/ |
| 19 | exp survival/ |
| 20 | exp prognosis/ |
| 21 | (Recurrence or Treatment failure or Disease Progression or Treatment Outcome* or Survival or Prognosis or disease progression or disease exacerbation or recurrent disease).tw,kw. |
| 22 | 14 or 15 or 16 or 17 or 18 or 19 or 20 or 21 |
| 23 | 4 and 7 and 13 and 22 |

**Cochrane Library**

ID Search Hits

#1 [mh "urinary bladder neoplasms"]

#2 [mh "carcinoma, transitional cell"]

#3 bladder cancer*:ti,ab,kw

#4 bladder tumor*:ti,ab,kw

#5 bladder tumour*:ti,ab,kw

#6 bladder neoplas*:ti,ab,kw

#7 urothelial cancer:ti,ab,kw

#8 urothelial carcinoma*:ti,ab,kw

#9 urothelial tumor*:ti,ab,kw

#10 urothelial tumour*:ti,ab,kw

#11 urothelial neoplas*:ti,ab,kw

#12 transitional cell carcinoma*:ti,ab,kw

#13 #1 or #2 or #3 or #4 or #5 or #6 or #7 or #8 or #9 or #10 or #11 or #12

#14 [mh "administration, intravesical"]

#15 bladder instillation therap*:ti,ab,kw

#16 bladder immune instillation therap*:ti,ab,kw

#17 intravesical instillation:ti,ab,kw

#18 intravesical chemotherap*:ti,ab,kw

#19 #14 or #15 or #16 or #17 or #18 1357

#20 [mh "female"] 603169

#21 [mh "male"] 569999

#22 [mh "sex"] 43

#23 [mh "women"] 1479

#24 [mh "men"] 102

#25 gender:ti,ab,kw 45468

#26 man:ti,ab,kw 91399

#27 men:ti,ab,kw 91399

#28 woman:ti,ab,kw 192489

#29 women:ti,ab,kw 192489

#30 male*:ti,ab,kw 914003

#31 female:ti,ab,kw 961906

#32 sex:ti,ab,kw 66083

#33 #20 or #21 or #22 or #23 or #24 or #25 or #26 or #27 or #28 or #29 or #30 or #31 or #32

#34 [mh "neoplasm recurrence, local"]

#35 [mh "recurrence"]

#36 [mh "treatment failure"]

#37 [mh "disease progression"]

#38 [mh "treatment outcome"]

#39 [mh "survival"]

#40 [mh "prognosis"]

#41 recurrence:ti,ab,kw

#42 recurrent disease:ti,ab,kw

#43 treatment failure:ti,ab,kw

#44 disease progression:ti,ab,kw

#45 treatment outcome*:ti,ab,kw

#46 survival:ti,ab,kw

#47 prognosis:ti,ab,kw

#48 disease progression:ti,ab,kw

#49 disease exacerbation:ti,ab,kw

#50 #34 or #35 or #36 or #37 or #38 or #39 or #40 or #41 or #42 or #43 or #44 or #45 or #46 or #47 or #48 or #49

#51 #13 and #19 and #33 and #50
